# The Severity of Carotid Calcifications, but Not Fibroblast Growth Factor 23, Is Associated with Mortality in Hemodialysis: A Single Center Experience

**DOI:** 10.3390/diseases13030073

**Published:** 2025-02-28

**Authors:** Diana Moldovan

**Affiliations:** Department of Nephrology, “Iuliu Hatieganu” University of Medicine and Pharmacy, 400012 Cluj-Napoca, Romania; diana.moldovan@umfcluj.ro

**Keywords:** carotid vascular calcification, fibroblast growth factor 23, hemodialysis, all-cause mortality, cardiovascular mortality

## Abstract

Background. The study goal was to assess the mortality effect of carotid vascular calcifications (VC), of fibroblast growth factor 23 (FGF-23), mineral markers, and comorbidities in hemodialysis (HD) patients. Methods. The influence of carotid VC severity, FGF-23, laboratory markers, clinical features, and comorbidities on mortality was analyzed in a cohort of 88 HD patients. The follow-up period lasted 8 years. The cut-off value for carotid VC was 4 for all-cause and cardiovascular mortality. Results. Carotid VC, diabetes, low serum albumin, high serum C-reactive protein (CRP), and the presence of cardiovascular diseases are associated with all-cause and cardiovascular mortality. Carotid VC score over 4 was an independent predictor of all-cause and cardiovascular mortality, along with diabetes, low albumin, and high CRP. FGF-23 was not found to be predictable for the study outcomes. Conclusions. The study documented in a cohort of patients prevalent in chronic HD that carotid VC predicts all-cause and cardiovascular mortality at 8 years and improves risk stratification, but FGF-23 is not associated with mortality. Other risk factors for all-cause and cardiovascular mortality were diabetes, inflammation, and malnutrition. However, future efforts are needed to assess whether a risk-based approach, including VC screening, improves survival.

## 1. Introduction

One can argue that people having end-stage renal disease (ESRD) are part of a privileged category compared with those with other vital organ failure, thanks to renal replacement therapies, which substantially prolong life. Nevertheless, it is estimated that chronic kidney disease accounts globally for 5 to 10 million deaths annually, mainly due to cardiovascular diseases [1]. However, there is a great need for improvement regarding the lifespan of these people. In 2016, chronic kidney disease was the sixteenth leading cause of years of life lost worldwide, and it is expected to be the fifth leading cause of years of life lost worldwide by 2040 [1]. Cardiovascular events account for the primary cause of mortality in hemodialysis (HD) patients.

Vascular disease can have an important mortality effect on patients with ESRD, and the risk for cardiovascular death is enhanced by the presence of atherosclerosis and vascular calcification (VC) [2]. Atherosclerosis is a complex and multifactorial process, characterized by early asymptomatic formation of plaque in the arterial walls, silent plaque progression and calcification that poses flow limitation, and risk of sudden thrombotic occlusion associated with a high rate of fatal evolution [2,3]. The early detection and grading of VC, or even better, of atherosclerotic plaque, and assessing the predictive value are critically important to prevent clinical consequences [3].

Vascular calcification (VC) is an active process analogous to bone formation and results not only from a passive deposition of calcium phosphate in the vessel walls but also from the activation of osteogenesis [2,4]. The differentiation of vascular smooth muscle cells into osteoblast-like cells [4] results in the synthesis of a bone structure in the arteries. Active inducers of VC in chronic kidney disease include hypercalcemia, hyperphosphatemia, abnormal iPTH levels, FGF-23, uremic toxicity, and inflammation [5]. The loss of mineralization inhibitors such as Klotho, pyrophosphates, and matrix gla protein leads to VC. Noteworthy are the systems of the osteoprotegerin (OPG)/RANK/RANK ligand complex and Wnt signaling pathway inhibitors such as sclerostin, which control bone matrix formation, including in the case of VC [4]. Besides classical risk factors for cardiovascular events, such as diabetes, dyslipidemia, and hypertension, kidney disease-specific risk factors, such as anemia, fluid overload, and chronic kidney disease—mineral bone disorders (CKD-MBD) contribute to this high mortality risk [5,6].

Extraskeletal calcifications represent the mineralization of the extracellular matrix. It is a condition frequently observed in the HD patient population, the main site for abnormal calcium salt deposition being the arteries [4,7]. The mechanisms underlying the pathogenesis of VC are complex, involving factors that facilitate or impede the development of calcification. Ongoing research has detected VC to be a cell-mediated process and a result of the combination of pro-calcifying stimuli and impairment of inhibiting mechanisms [7]. Intimal calcification complicating atherosclerosis and medial calcification are both possible consequences of ESRD that can occur simultaneously [8]. Endothelial cells exposed to various pro-calcification factors are injured, and they easily become prone to VC. Extended research brought evidence in favor of the pathogenetic pathway of VC, explained by the fact that endothelial cells are not simple bystanders, being able to undergo a phenotypic switch and become bone progenitor cells to promote calcification [9]. The crosstalk between vessels and bone is a continuum in VC occurrence, with all the above-mentioned factors acting on arteries and bones consecutively [10]. Arterial calcification and elevated FGF-23 levels may be significantly involved in the development of heart conditions such as left ventricular hypertrophy, coronary ischemic disease, and consequent congestive heart failure in chronic kidney disease, predisposing factors for early cardiovascular death [11].

The role of calcification inhibitors and regulators in the calcification process, as well as their effect on vascular dysfunction and mortality in HD patients, is not completely understood. Fibroblast growth factor 23 (FGF-23) is an important regulator of mineral homeostasis [12] and is a circulating hormone primarily released by bone, produced in osteocytes. Circulating FGF-23 stimulates urinary phosphate excretion and inhibits the production of active 1,25-dihydroxy vitamin D [13]. Serum FGF-23 levels increase early during chronic kidney disease progression before changes in other parameters of bone and mineral metabolism. Several studies have reported that a higher level of FGF-23 is associated with an increased risk of death in patients with ESRD [14] or with other adverse outcomes such as the progression of diabetic nephropathy [15]. No consensus has been obtained yet regarding the role of FGF-23 in the evolution of HD patients.

There are some theories that outcomes for ESRD patients are different according to findings related to CKD-MBD, such as serum levels of FGF-23 and the presence or severity of vascular calcifications; however, it is generally hypothesized that the benefits observed with early interventions depend on how early risk is detected and addressed [11]. More importantly, there is currently no efficient treatment for VC, so understanding the impact on outcome and prevention is crucial.

The primary aim of this study was to investigate the association between carotidal atheroma VC, serum FGF-23 levels, and all-cause mortality in chronic HD patients. The secondary goals were to investigate the causes of death and the impact of different demographical, clinical, and laboratory factors on all-cause and cardiovascular mortality.

## 2. Materials and Methods

This is a prospective, longitudinal, and analytical study. The study population consisted of a group of 88 patients treated with chronic hemodialysis. Eligibility criteria: adult patients over 18 years old, prevalent in dialysis, who accepted the study protocol. Exclusion criteria: life expectancy less than 6 months, previous parathyroidectomy, previous renal transplant, active infections. Demographic and clinical data (age, gender, HD vintage, HD prescription, treatments with Ca-based phosphates (P) binders, sevelamer, and vitamin D, presence of diabetes and hypertension, cardiovascular diseases). Laboratory evaluation included serum calcium (Ca), inorganic phosphorus (inorganic P), alkaline phosphatase (ALP), intact parathyroid hormone (iPTH), bicarbonate, creatinine, hemoglobin (Hb), ferritin, albumin, and C-reactive protein (CRP); fibroblast growth factor 23 (Human FGF-23; ELISA). Hemodialysis adequacy was presented using spKt/V and urea reduction ratio (URR).

**Figure 1:**
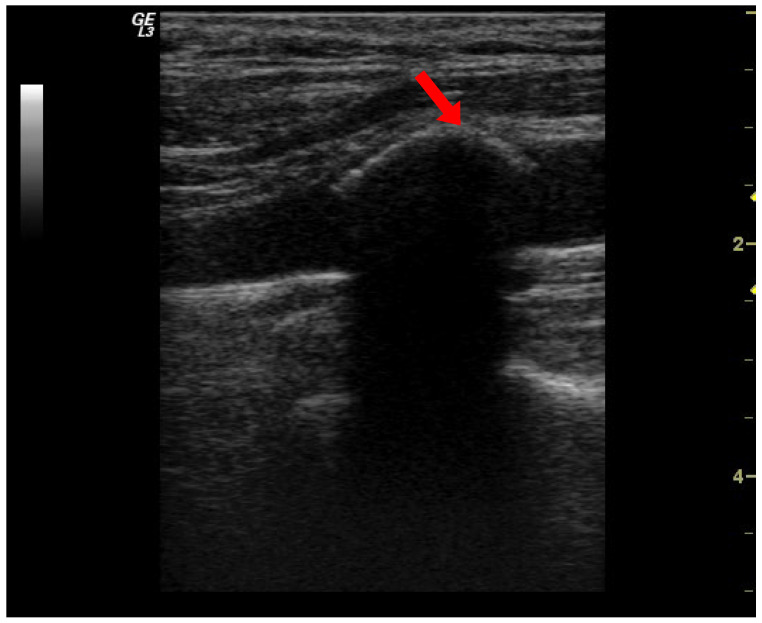
Ultrasound examination. Common carotid artery calcification is the patchy hyperechoic image with a posterior shadow in the arterial wall (red arrow).

Ultrasound examinations have been performed on three segments of the carotid arteries (bilateral): common carotid artery, bifurcation, and internal carotid artery. Real-time and color Doppler sonography through longitudinal and transversal sections were undertaken. Two exams assured the intra-observer reproducibility of this assessment; in selected cases, a third examination was performed. Carotid vascular calcifications (VC) were defined as patchy hyperechoic images with posterior shadows in the arterial walls, representing calcified atheroma plaques. A VC score was counted ranging from 0 (no calcification) to 6 (calcification of all artery sites examined from both sides); each examined site received 1 point in this score (Figure 1).

Cardiovascular diseases were registered and included ischemic heart disease, heart failure, stroke, arrhythmia, aortic aneurysm, and peripheral artery disease.

All 88 included HD patients had a standard HD schedule of 3 sessions of 4 h/week and achieved a mean spKt/V of 1.53 ± 0.28. They had a mean age of 59.68 ± 14.49 years, and a mean HD vintage of 59.61 ± 52.39 months. Forty-five were males (51.13%), 63 patients (71.6%) had hypertension, 20 patients (22.72%) had diabetes mellitus, 54 patients (61.4%) had carotidal VC, and 25 patients (28.4%) had cardiovascular diseases. The median FGF-23 was 43.5, ranging from 7.6 to 290.8 pg/mL. The patients’ characteristics are described in detail in our previous cross-sectional study [16].

The follow-up period lasted 8 years. This study recorded the mortality causes and survival time. Mortality data were obtained from the dialysis center register. The cardiovascular causes of death included myocardial infarction, heart failure, arrhythmia, pulmonary edema, stroke, aortic aneurysm, and peripheral artery disease. Factors with impact on all-cause and cardiovascular death were analyzed.

### Statistical Analysis

Normally distributed data are presented as mean and standard deviation (SD) and skewed variables as median and interquartile range. Normality was assessed using Kolmogorov–Smirnov and Shapiro–Wilk tests. Categorical variables are shown as total numbers and percentages. Comparison between groups was performed by the Chi-square test, Fisher exact test, independent samples *t*-test, and Mann-Whitney test as appropriate. Univariate and multivariate Cox proportional hazard models were used to estimate the association between independent predictors and all-cause and cardiovascular mortality in all patients. The multivariable model included potential confounders influencing mortality, which became significant in univariate analysis. Cox proportional hazard models were used to estimate hazard ratios (HR) for the outcome of interest described above. The receiver-operating characteristic (ROC) curve identified the cut-off value of carotidal VC that best-predicted all-cause and cardiovascular deaths. To visualize significant associations between VC score (<4 vs. ≥4) and mortality, Kaplan–Meier curves were generated; these were compared by the log-rank test.

Statistical analyses were performed using SPSS version 25.0 (IBM, Armonk, NY, USA). For all tests, a two-sided *p*-value < 0.05 was regarded as statistically significant.

## 3. Results

Death of all causes was recorded and analyzed. In our study group, forty-nine patients died (55.7%) for the entire follow-up period. Among all patients included in the study, 34 patients died due to cardiovascular causes (38.6%). Cardiovascular mortality was the consequence of myocardial infarction in 8 patients, heart failure in 7 patients, arrhythmia in 5 patients, pulmonary edema in 4 patients, stroke in 5 patients, and peripheral artery disease in 5 patients. The other patients died due to infections (7 patients), neoplasia (2 patients), sudden death (3 patients), or unknown cause (3 patients).

The patients were included in 2 categories, deceased and survivors, and compared them. It resulted in a significant association between all-cause mortality and older age, male gender, more severe carotidal VC, increased CRP, low albumin, presence of diabetes, and cardiovascular diseases (Table 1).

Univariate Cox proportional hazards regression analysis for all-cause and cardiovascular mortality in HD patients is detailed in Table 2. The results of univariate regression demonstrated that advanced age (*p =* 0.002), diabetes (*p =* 0.001), carotidal VC (*p <* 0.001), increased CRP (*p <* 0.001), decreased albumin (*p =* 0.002) and presence of cardiovascular diseases (*p <* 0.001) are significantly associated with high risk of all-cause mortality. Serum FGF-23 levels had no significant impact on all-cause (*p =* 0.67) or cardiovascular mortality (*p =* 0.84) (Table 2). The analysis of cardiovascular mortality in univariate regression demonstrated a significant association with increased age (*p =* 0.019), increased carotidal VC score (*p =* 0.003), low URR and spKt/V (*p =* 0.02), high CRP (*p <* 0.001), presence of diabetes (*p =* 0.01) and cardiovascular diseases (*p =* 0.013) (Table 2).

The study evaluated how the severity of carotidal VC can predict mortality. The cut-off value for the VC score to predict all-cause mortality was 4. The area under the ROC curve was 0.718 (*p* < 0.001; 95% CI = 0.609–0.826). The sensitivity was 55.1% and the specificity was 85%.

The cut-off value for VC score which predicts cardiovascular mortality was also 4. The area under the ROC curve was 0.657 (*p* = 0.014; 95% CI = 0.539–0.775). The sensitivity was 55.9% and the specificity was 74.1%.

**Figure 2:**
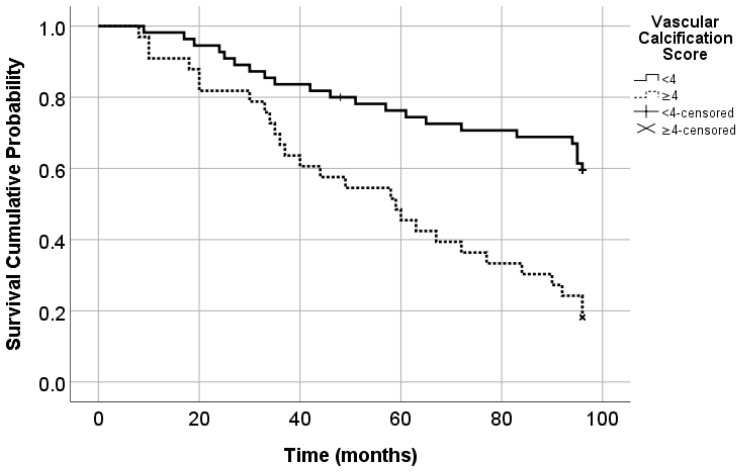
All-cause mortality according to the VC score (Log Rank Mantel Cox; *p* < 0.001). Survival probability at 96 months was 60% for patients with VC score < 4 and 19.2% for patients with VC score < 4.

The group with a VC score < 4 consisted of 55 patients and 22 deaths (mortality rate = 40%), and the group with a VC score ≥ 4 consisted of 33 patients (37.5%) with 27 deaths (mortality rate = 81.8%) (Figure 2).

**Figure 3:**
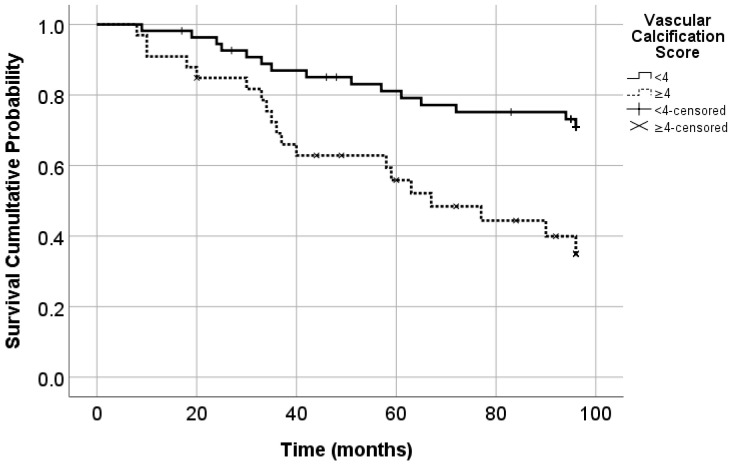
Cardiovascular mortality according to the VC score (Log Rank Mantel Cox; *p* = 0.005). Cardiovascular events produced death in 27.3% of patients with VC score < 4 and in 57.6% of patients with VC score ≥ 4.

In 6 patients from the survivors’ group (15.4%) and in 27 patients from the deceased group (55.1%), the VC score was ≥4 (*p* < 0.001). The Kaplan-Meyer analysis showed that from the group of 55 patients with VC score < 4, 27.3% died due to cardiovascular causes, and from the group of 33 patients with VC score ≥ 4, 57.6% died due to cardiovascular causes (*p* = 0.005) (Figure 3).

Multivariate Cox regression analyzed the influence of carotid VC on mortality. All-cause mortality was associated with carotid VC score, diabetes, and CRP; covariates were all significant factors in univariate analysis and factors relevant to patients’ outcomes. No significant relation was found between FGF-23 and all-cause mortality (Table 3).

Cardiovascular mortality was also associated with carotid VC score, diabetes, and CRP, but also with HD adequacy assessed by URR; covariates were significant factors in univariate analysis (Table 4).

## 4. Discussion

A high prevalence of VC of the carotid arteries on ultrasound examinations was identified. The arguments in favor of vascular ultrasound evaluations include easy access to devices, a high rate of use in most medical facilities, a large number of physicians trained to perform echography, its non-invasive nature, and safety with no risk added. Numerous studies validated the ability of arterial ultrasound examinations to properly assess the presence and extent of calcifications and similar results were reported by other authors as well [3,16,17]. A recent study demonstrated that the ultrasound-based method correlates with standard computer tomography (CT)--based methods for femoral artery evaluation. Ultrasound-based calcification scores were increased in patients with diabetes, renal failure, and the presence of chronic limb ischemia similar to CT-based femoral calcification [17]. Due to the reliability of the method, in recent years, a higher interest extending even to intravascular ultrasound in specialized centers is acknowledged. The aim of the latest is to detect vascular wall microcalcification because the mechanical stresses are augmented, and the clinical risk occurs from the early stages of intimal calcium formation [18,19]. Nevertheless, it is generally accepted that the use of common ultrasound by skilled clinicians improves vascular assessment with reasonable accuracy, and by now, this is widely available.

The present study identified a strong, significant association of carotid calcified atheroma plaques with all-cause and cardiovascular mortality. Such correlations between VC and mortality were reported by numerous studies. Coronary artery calcification detected on CT exams increased mortality in patients undergoing hemodialysis [20]. In peritoneal dialysis patients, it was reported that discrete modifications of arteries, such as carotid intima-media thickness, are associated with cardiovascular mortality [21]. The INDEPENDENT study checked for the power of death prediction in incident HD patients of VC assessed with coronary artery calcification Agatston score and with abdominal aorta calcification Kauppila score. Each type of VC predicted all-cause mortality in this study [3]. Cardiovascular risk can also be stratified by assessing the atheroma through ultrasound of arteries [22].

Chang et al. evaluated the aortic arch calcifications of HD patients on chest X-rays using a three-grade scoring of severity. The patients with coexisting moderate-to-severe aortic arch calcification and high alkaline phosphatase had a higher risk of major cardiovascular events, and cardiovascular and all-cause mortality compared to those with non-to-mild aortic arch calcification/low alkaline phosphatase even after adjustments for significant clinical variables. Authors concluded that moderate-severe aortic arch calcification and high serum alkaline phosphatase co-modify the risk of cardiovascular events and mortality among chronic HD patients at 3 years of follow-up [23].

Regarding cardiovascular diseases in the present study, they were associated with all-cause and cardiovascular mortality in univariate analysis; this effect was lost after multivariate analysis. Further larger studies are needed to clarify this relationship.

Low and high PTH and hyperphosphatemia as well facilitate VC. The FGF 23-Klotho axis has also been implicated in VC. FGF-23, a bone-derived hormone maintaining phosphate balance, has emerged as a key player in CKD-MBD pathophysiology. This study did not find any significant relationship between serum FGF-23 levels and all-cause mortality or cardiovascular mortality. A previous report about FGF-23 effects in HD patients demonstrated an association between elevated FGF-23 levels and death [24]. FGF-23 was also associated with death in non-dialysis chronic kidney disease stages 3–5 patients [25]. In a recent report on the EVOLVE trial, FGF-23 was a risk factor for cardiovascular calcification, events, and mortality in HD patients [26,27]. Conversely, our prior cross-sectional study demonstrated that low FGF-23 is associated with the presence of cardiovascular diseases [16]. No relationship between FGF-23 and atherosclerosis, arterial stiffness, and peripheral vascular complications was reported by other studies [28]. In the PREVEND study, on a prospective population-based cohort, high FGF-23 levels were associated with an increased risk of new-onset chronic kidney disease and all-cause mortality, independent of established chronic kidney disease risk factors [29]. However, Olauson et al. [30] and Mizuiri [20] reported no association between high serum FGF-23 and mortality in HD patients, which is consistent with our findings. PACE study reached the same conclusion that FGF-23 is not associated with death [31]. Bouma de Krijger et al. tested the change in FGF-23 concentration over time and its association with all-cause mortality in patients treated with HD or hemodiafiltration. The results of their study, named CONTRAST, was that there is not any association between a single value of FGF-23 and all-cause mortality, but increasing FGF-23 concentrations did identify patients at risk for mortality. No association was found between baseline FGF-23 concentrations and all-cause mortality among prevalent dialysis patients, and this is also a similarity with my study results. CONTRAST study also confirmed that hemodiafiltration is capable of substantially reducing plasma FGF-23 concentrations. However, since lowering FGF-23 did not improve outcomes, this study found no argument for therapeutically lowering FGF-23 [32]. The CONTRAST study results may help us find an explanation for the lack of correlation between serum FGF-23 levels and mortality. The possibility that high-flux filters used to dialyze our patients could have contributed to FGF-23 clearance, influencing the serum levels, and consequently, the study’s results [32]. Further studies may bring clarity regarding this hypothesis.

Carotid intima-media thickness, FGF-23, and mineral bone disorder were analyzed in a cross-sectional study on 42 children aged 2–18 years old with chronic kidney disease stages 2 to 5D. The study has shown that FGF-23 levels increase with chronic kidney disease progression, but there were no significant correlations between carotid intima-media thickness and factors, including mineral markers and FGF-23 levels [33]. The discrepancies between the results of these studies might be at least partially explained by age, lifestyle, and racial differences and also by the HD prescription, efficiency, and even ultrafiltration [34,35]. In conclusion, in prevalent HD patients, baseline FGF-23 value is not associated with all-cause mortality, suggesting that the association between FGF-23 and long-term outcome may disappear with dialysis duration. Furthermore, low FGF-23 concentrations are not associated with a lower cardiovascular risk [16]. This observation argues against the benefit of interventions that lower FGF-23 in prevalent patients with HD.

Diabetes mellitus proved to be a risk factor for all-cause and cardiovascular mortality in ESRD patients. This is a traditional risk factor acknowledged by different studies [36]. Low serum albumin levels were linked to all-cause and also to cardiovascular mortality, emphasizing the role of malnutrition and inflammation in the outcome of HD patients [37]. There is an ongoing interest in prediction models for mortality. A new construction proved that a C-reactive protein–albumin–lymphocyte could be used as a prediction model for all-cause mortality in patients on maintenance hemodialysis, emphasizing the role of serum albumin and CRP in the enhanced risk of death [38]. In our study, serum CRP levels were identified as a strong predictor for all-cause and cardiovascular mortality. Inflammation was outlined in ongoing research as a risk factor for negative outcomes in HD patients [8,39,40], including death [37,41]. A recent study conducted on a cohort of 3262 participants from the US National Health and Nutrition Examination Survey (NHANES) database proved that the systemic inflammatory response is associated with all-cause mortality and cardiovascular mortality in a population with chronic kidney disease. The systemic inflammatory response independently posed a risk for both all-cause and cardiovascular mortality in chronic kidney disease patients [42]. In our study, cardiovascular mortality was also influenced by HD adequacy, low URR increased the death risk by cardiovascular causes.

As a validation of previous studies, the recent KDIGO guideline for the management of chronic kidney disease and the conclusions of the newest controversial conference on CKD-MBD recognize diabetes, malnutrition inflammation, and VC as risk factors for premature death in chronic kidney disease [43].

The primary strength of this study was the long follow-up period with reliable and detailed information. However, there are several potential limitations. The relatively small sample size is the first and probably the most relevant one. Secondly, variables related to cardiovascular risks, such as cardiac echogram parameters, smoking habits, and physical exercise, were not included in this study. Finally, these results were obtained from a single center, and the patients were all Caucasians, so the results might not be applied to all hemodialysis people. The ultrasound-based method shows promise as a simple method for quantifying the extent of carotid artery calcification in patients with ESRD. The correlation with mortality shows that it could be useful for predicting outcomes for HD patients, extending access to VC screening.

## 5. Conclusions

The study documented in a cohort of patients prevalent in chronic HD that carotid VC predicts all-cause and cardiovascular mortality at 8 years of follow-up and significantly improves risk stratification. FGF-23 was not associated with outcomes. Other significant risk factors for all-cause and cardiovascular mortality were diabetes, high CRP, and low albumin. HD adequacy influenced cardiovascular death risk. In conclusion, our findings emphasize that the adverse prognosis associated with carotid VC in ESRD is exacerbated by the severity and extent of calcifications, encompassing age, diabetes, or inflammation. Conversely, normal serum albumin and an adequate HD may offer protective effects on carotid VC-related poor outcomes, potentially through mechanisms involving nutrition, elimination of uremic toxins, or anti-inflammatory pathways. Therefore, this study illustrates the concept of multiple pathogenic hits contributing to the high risk of death in HD patients. Consequently, effective therapeutic strategies aimed at improving outcomes in HD patients should focus on mitigating the harmful risk factors, including chronic inflammation, malnutrition, and normalizing mineral metabolism and vascular bone remodeling. However, future efforts are needed to assess whether a risk-based approach, including VC screening to guide the management of chronic HD patients improves survival.

## Figures and Tables

**Table 1:** Comparison between the deceased and survivor groups.

	Deceased (49 Patients)	Survivors (39 Patients)	*p*
Age (years)	63 (59–72)	56 (46–64)	**0.003**
HD vintage (months)	47 (29–64)	49 (29.5–68)	0.628
Gender, males no. (%)	27 (55.1)	18 (46.2)	0.404
Diabetes, no. (%)	17 (34.7)	3 (7.7)	**0.003**
HTN, no. (%)	38 (77.6)	25 (64.1)	0.165
FGF-23 (pg/mL)	42.30 (23.10–72.70)	41.70 (20.95–70.90)	0.592
VC score	4 (1–6)	0 (0–2)	**<0.001**
URR	72.32 (69.57–79.45)	77.33 (69.63–80.65)	0.183
spKt/V	1.46(1.39–1.63)	1.58 (1.40–1.66)	0.183
Bicarbonate (mEq/L)	24 (22.7–25.6)	22.9 (20.5–24.6)	0.084
K (mEq/L)	4.48 ± 0.64	4.72 ± 0.55	0.067
Ca (mg/dL)	9.09 ± 0.65	9.07 ± 0.67	0.898
P (mg/dL)	4.34 ± 1.10	4.57 ± 1.54	0.427
ALP (U/L)	78.68 (64.13–99.56)	67.75 (45.22–103.84)	0.957
iPTH (pg/mL)	212.25 (144.75–547.65)	273.4 (160.95–536)	0.332
Hb (g/dL)	11.3 (10.6–12)	11.4 (10.5–12.35)	0.072
Ferritin (ng/mL)	498.51 (303.45–791.35)	586.22 (402.46–816.45)	0.784
CRP (mg/dL)	0.80 (0.35–2.02)	0.47 (0.28–1.04)	**0.035**
Albumin (g/dL)	3.75 (3.57–3.94)	3.96 (3.80–4.08)	**0.005**
Creatinine (mg/dL)	8.20 ± 1.98	8.73 ± 2.53	0.265
Ca in HD solution	1.5 (1.25–1.5)	1.5 (1.25–1.5)	0.854
Treatment with Ca salts, no. (%)	30 (61.2)	17 (43.6)	0.099
Sevelamer, no. (%)	10 (20.4)	10 (25.6)	0.561
Vitamin D Treatment, no. (%)	10 (20.4)	10 (25.6)	0.561
Cardiovascular diseases, no. (%)	23 (46.9)	4 (10.3)	**<0.001**

Legend: FGF-23, fibroblast growth factor 23; HD, hemodialysis; HTN, arterial hypertension; VC, vascular calcification; URR, urea reduction ratio; spKt/V, dialysis adequacy; K, kalium; Ca, calcium; P, phosphorus; ALP, alkaline phosphatase; iPTH, intact parathyroid hormone; Hb, hemoglobin; CRP, C-reactive protein. Data are expressed as mean ± standard deviation and as median (25th–75th percentiles) or percentages. Statistical significance is marked with bold characters.

**Table 2:** Results of univariate Cox regression analysis for all-cause and cardiovascular mortality.

	All-Cause Mortality		Cardiovascular Mortality	
	HR (95% CI)	*p*	HR (95% CI)	*p*
Age (years)	1.04 (1.01–1.06)	**0.002**	1.03 (1.00–1.06)	**0.019**
HD vintage (months)	0.99 (0.99–1.00)	0.577	0.99 (0.99–1.00)	0.785
Gender (males)	0.77 (0.43–1.35)	0.361	0.67 (0.34–1.33)	0.254
Diabetes	2.74 (1.50–5.01)	**0.001**	2.56 (1.25–5.24)	**0.01**
HTN	1.41 (0.72–2.77)	0.307	1.11 (0.52–2.39)	0.779
FGF-23 (pg/mL)	1.00 (0.99–1.00)	0.676	1.00 (0.99–1.00)	0.841
Carotid VC score	1.24 (1.11–1.39)	**<0.001**	1.22 (1.07–1.40)	**0.003**
URR	0.98 (0.96–1.00)	0.119	0.97 (0.95–0.99)	**0.026**
spKt/V	0.48 (0.19–1.20)	0.119	0.33 (0.12–0.87)	**0.026**
Bicarbonate (mEq/L)	1.07 (0.98–1.16)	0.092	1.05 (0.95–1.16)	0.342
K (mEq/L)	0.65 (0.39–1.06)	0.085	1.03 (0.58–1.81)	0.917
Ca (mg/dL)	1.01 (0.64–1.60)	0.948	1.27 (0.73–2.23)	0.395
P (mg/dL)	0.88 (0.71–1.09)	0.263	0.89 (0.69–1.16)	0.411
ALP (U/L)	1.00 (0.99–1.00)	0.945	1.00 (0.99–1.00)	0.590
iPTH (pg/mL)	1.00 (0.99–1.00)	0.210	1.00 (0.99–1.00)	0.399
Hb (g/dL)	0.85 (0.70–1.04)	0.117	0.89 (0.71–1.13)	0.375
Ferritin (ng/mL)	1.00 (0.99–1.00)	0.788	1.00 (1.00–1.00)	0.514
CRP (mg/dL)	1.24 (1.10–1.38)	**<0.001**	1.27 (1.13–1.44)	**<0.001**
Albumin (g/dL)	0.41 (0.23–0.71)	**0.002**	0.58 (0.27–1.24)	0.162
Creatinine (mg/dL)	0.95 (0.84–1.06)	0.371	1.03 (0.89–1.19)	0.660
Ca in HD solution	1.03 (0.67–1.58)	0.890	0.79 (0.45–1.30)	0.330
Treatment with Ca salts—patients (%)	1.50 (0.84–2.68)	0.164	1.17 (0.59–2.31)	0.644
Sevelamer—patients (%)	0.69 (0.34–1.39)	0.303	0.71 (0.31–1.64)	0.430
Vitamin D Treatment—patients (%)	0.75 (0.37–1.50)	0.419	0.76 (0.33–1.75)	0.521
Cardiovascular diseases	2.84 (1.61–5.01)	**<0.001**	2.38 (1.20–4.72)	**0.013**

Legend: HR, hazard ratio; CI, confidence interval; FGF-23, fibroblast growth factor 23; HD, hemodialysis; HTN, arterial hypertension; VC, vascular calcification; URR, urea reduction ratio; spKt/V, dialysis adequacy; K, kalium; Ca, calcium; P, phosphorus; ALP, alkaline phosphatase; iPTH, intact parathyroid hormone; Hb, hemoglobin; CRP, C-reactive protein. *p* < 0.05 was statistically significant and was marked with bold.

**Table 3:** Multivariate Cox regression analysis for all-cause mortality.

	All-Cause Mortality	
	HR (95% CI)	*p*
Age (years)	1.02 (0.99–1.05)	0.185
FGF-23 (pg/mL)	1.00 (0.99–1.00)	0.090
Diabetes	2.35 (1.19–4.64)	**0.014**
VC score	1.19 (1.01–1.39)	**0.031**
K	0.94 (0.51–1.73)	0.860
Bicarbonate	1.09 (0.97–1.23)	0.122
Albumin	0.66 (0.31–1.43)	0.297
CRP	1.28 (1.12–1.46)	**<0.001**
Cardiovascular diseases	1.34 (0.67–2.68)	0.400

Legend: HR, hazard ratio; CI, confidence interval; FGF-23, fibroblast growth factor 23; VC, vascular calcification; K, kalium; CRP, C-reactive protein. A value of *p* < 0.05 marked statistical significance and it was highlighted with bold.

**Table 4:** Multivariate Cox regression analysis for cardiovascular mortality.

	Cardiovascular Mortality	
	HR (95% CI)	*p*
Age (years)	1.02 (0.98–1.06)	0.221
FGF-23 (pg/mL)	1.00 (0.99–1.00)	0.432
Diabetes	2.16 (1.03–4.55)	**0.041**
VC score	1.22 (1.02–1.47)	**0.028**
CRP	1.36 (1.18–1.56)	**<0.001**
URR	0.95 (0.93–0.99)	**0.009**
Cardiovascular diseases	1.12 (0.51–2.44)	0.762

Legend: HR, hazard ratio; CI, confidence interval; FGF-23, fibroblast growth factor 23; VC, vascular calcification; CRP, C-reactive protein; URR, urea reduction ratio. A value of *p* < 0.05 marked statistical significance and it was highlighted with bold.

## Data Availability

Data are contained within the article.
